# The male-to-female ratio in late-onset multiple acyl-CoA dehydrogenase deficiency: a systematic review and meta-analysis

**DOI:** 10.1186/s13023-024-03072-6

**Published:** 2024-02-16

**Authors:** Jing Ma, Huiqiu Zhang, Feng Liang, Guanxi Li, Xiaomin Pang, Rongjuan Zhao, Juan Wang, Xueli Chang, Junhong Guo, Wei Zhang

**Affiliations:** 1 Department of Neurology, First Hospital, Shanxi Medical University, No.85, Jiefang South Street, Taiyuan, China; 2 First Clinical Medical College, Shanxi Medical University, Taiyuan, China

**Keywords:** Late-onset multiple acyl-CoA dehydrogenase deficiency, Male-to-female ratio, Sex difference, Meta-analysis, Ethnicity

## Abstract

**Background:**

Late-onset multiple acyl-CoA dehydrogenase deficiency (MADD) is the most common lipid storage myopathy. There are sex differences in fat metabolism and it is not known whether late-onset MADD affects men and women equally.

**Methods:**

In this systematic review and meta-analysis, the PubMed, Embase, Web of Science, CNKI, CBM, and Wanfang databases were searched until 01/08/2023. Studies reporting sex distribution in patients with late-onset MADD were included. Two authors independently screened studies for eligibility, extracted data, and assessed risk of bias. Pre-specified outcomes of interest were the male-to-female ratio (MFR) of patients with late-onset MADD, the differences of clinical characteristics between the sexes, and factors influencing the MFR.

**Results:**

Of 3379 identified studies, 34 met inclusion criteria, yielding a total of 609 late-onset MADD patients. The overall pooled percentage of males was 58% (95% CI, 54-63%) with low heterogeneity across studies (*I*^*2*^ = 2.99%; *P* = 0.42). The mean onset ages, diagnostic delay, serum creatine kinase (CK), and allelic frequencies of 3 hotspot variants in *ETFDH* gene were similar between male and female patients (*P* > 0.05). Meta-regressions revealed that ethnic group was associated with the MFR in late-onset MADD, and subgroup meta-analyses demonstrated that East-Asian patients had a higher percentage of male, lower CK, and higher proportion of hotspot variants in *ETFDH* gene than non-East-Asian patients (*P* < 0.05).

**Conclusions:**

Male patients with late-onset MADD were more common than female patients. Ethnicity was proved to be a factor influencing the MFR in late-onset MADD. These findings suggest that male sex may be a risk factor for the disease.

**Supplementary Information:**

The online version contains supplementary material available at 10.1186/s13023-024-03072-6.

## Introduction

Multiple acyl-CoA dehydrogenase deficiency (MADD), also known as glutaric acidemia type II (GAII), is an autosomal recessive disease that affects fatty acid, amino acid, and choline metabolisms [1]. The clinical phenotype of MADD can be classified as neonatal-onset forms with or without congenital anomalies and late-onset form [1]. Late-onset MADD is the most common lipid storage myopathy, predominantly caused by defects in electron transfer flavoprotein dehydrogenase (ETFDH) [1, 2]. The *ETFDH* gene encodes ETFDH, which participates in the process of fatty acid β-oxidation in the mitochondrial matrix. Electron transfer flavoprotein (ETF) genes (*ETFA* and *ETFB*), some riboflavin transporter genes (*SLC52A1*, *SLC52A2*, *SLC52A3*, and *SLC25A32*), and FAD synthase gene (*FLAD1*) were also reported as the causative genes for late-onset MADD [3, 4].

Although late-onset MADD is an inherited disease, several factors in addition to genetic mutations contribute to the disease. Riboflavin deficiency is common in late-onset MADD patients before treatment, in plasma and muscles [5, 6], and plays a crucial role in the pathogenesis of late-onset MADD [5]. The homozygous knock-in mice (*Etfdh*^*(h)A84T*^*/*^*(h)A84T*^), as a model of late-onset MADD, developed the disease, when fed by riboflavin deficient and high fat diet [5]. Moreover, metabolic stress is essential to the disease onset, such as cold, excessive physical training, infection, fasting, and using antiobesity drugs [2, 5, 7–10].

Sex differences are caused by genetic and gonadal hormone influences on epigenetics, gene expression and metabolism at the cellular and systems levels [11]. Many diseases affect males and females differently [12]. For example, men have a higher risk of amyotrophic lateral sclerosis than women [13]. It came to our attention that male patients outnumbered female patients in our cohort of late-onset MADD with a male-to-female ratio (MFR) of 8:5 in the context of that autosomal recessive disorders usually affect men and women equally [6, 14]. Most other studies also revealed late-onset MADD patients were more common in male than female patients [2, 5, 8, 9, 15–17], although an opposite trend was observed in a few cohorts [18–20]. As there are pronounced differences in fat metabolism between men and women [21], male sex may be a risk factor of the disease. However, the MFR of late-onset MADD has not been ascertained because of a small sample size and conflicting results of the previous studies. A meta-analysis provides greater statistical power. Therefore, the present study aims to identify the MFR of late-onset MADD.

## Methods

We followed the Preferred Reporting Items for Systematic Reviews and Meta-analyses (PRISMA) reporting guidelines [22] to conduct a systematic review (Supplementary Table 1). The study protocol was registered and published on the International Prospective Register of Systematic Reviews (CRD42023458620).

### Eligibility criteria

All studies reporting MADD were identified. Studies reporting sex distribution of late-onset MADD patients were included. Case reports, case series of less than 5 late-onset MADD patients, systematic reviews, conference abstracts, and animal studies were excluded. If patients overlapped in two studies, the one with a smaller sample size was excluded. The primary outcome was the percentage of male patients with late-onset MADD. Other outcomes of interest included the differences of mean onset ages, diagnostic delay, serum creatine kinase (CK) at diagnosis, and allelic frequencies of 3 hotspot variants in *ETFDH* gene (c.250G > A, c.770 A > G, and c.1227 A > C) [2, 8] between the sexes, and factors influencing the MFR, including ethnic groups, risk of bias, onset age, the MFR of general population of countries and the HDI of countries.

### Search methods

A systematic search was conducted on 01/08/2023 using 6 databases including PubMed, Embase, Web of Science, China National Knowledge Infrastructure (CNKI), Chinese Biomedical Literature Database (CBM), and Wanfang database, to identify any relevant study. The search strategy used the terms “multiple acyl-CoA dehydrogenase deficiency” or “glutaric aciduria II” or “glutaric acidemia II”. Detailed search strategies are available in the Appendix S1. Retrieved studies were imported into Covidence, a web-based software platform designed to support citation screening and collaboration among multiple authors.

### Study selection

After removal of duplicate studies, two reviewers (J.M. and H.Q.Z.) screened titles and abstracts independently. Next, the full texts of potentially relevant articles were read for eligibility according to the inclusion and exclusion criteria. Reasons for excluding studies at full-text review were recorded. Any disagreement was resolved by discussion with a senior author (W.Z.) to achieve consensus. Our retrospective study fulfilled the eligibility criteria and was included in the meta-analyses, although it hadn’t been published until 01/08/2023 [6]. A flow diagram was used to show the process of study selection following the PRISMA guidelines.

### Data collection

A standardized data extraction form was developed (available on request). Two reviewers (J.M. and H.Q.Z.) extracted and checked data independently for included articles, including name of the first author, year of publication, study design, study period, ethnic group, number and type (tertiary or not) of centers included, number of patients with late-onset MADD, number of male and female patients, onset age, diagnostic delay, serum CK at diagnosis, and allelic frequencies of the 3 hotspot variants in *ETFDH* gene, if reported.

### Assessment of bias

Two reviewers (J.M. and H.Q.Z.) independently assessed the risk of bias on each article using the Joanna Briggs Institute (JBI) Critical Appraisal Checklist for case series [23]. The final score of each article was calculated based on the percentage of positive answers (“yes”) and the risk of bias was ranked as high (score ≤ 49%), moderate (score ranging from 50 to 69%), and low (score ≥ 70%) [24].

### Data synthesis and analysis

Statistical analyses were performed using the statistical software STATA 16.0 (StataCorp LP, College Station, TX, USA). We used a meta-analysis with a DerSimonian and Laird random-effects model, based on a proportions approach, to determine the percentage of male patients with late-onset MADD. Weighted *t* tests using a random-effects model (DerSimonian and Laird method) were conducted to compare the weighted means of onset age, diagnostic delay, and serum CK at diagnosis between male and female patients. To compare the allelic frequencies of the 3 hotspot variants in *ETFDH* gene between the sexes, odds ratio (OR) meta-analyses were done using a random-effects model (DerSimonian and Laird method) separately. The pooled estimates were presented together with the associated 95% confidence intervals (CIs) using forest plots. The *I*^*2*^ statistic and Cochran’s *Q* tests were used to measure the between-study heterogeneity and a value of *I*^*2*^ > 50% or *P* < 0.05 for the *Q* test was considered as significant heterogeneity [25]. Meta-regression analyses were implemented separately to investigate influences on the MFR of late-onset MADD. The tested moderators included risk of bias, ethnic group (East-Asians or non-East-Asians), onset age, MFR of general population of country of origin (https://cn.knoema.com/), and UN’s 2022 Human Development Index (HDI) of country of origin (http://hdr.undp.org/en/data). When a significant association was found, we checked for potential confounders and controlled for these by multivariable meta-regression. If a significant moderator was detected, subgroup analyses were performed using the meta-analytic procedures described above. Considering the sex hormones which have important effect on metabolism vary greatly across age [26], subgroup meta-analysis through stratifying patients by the median of the average onset ages of included studies was also performed. The average onset age for each study was estimated by taking a median onset age of patients. The funnel plot and Egger regression tests were used to test publication bias. Sensitivity analyses were planned for the primary outcome by: (1) excluding studies with a JBI score less than 90%, (2) excluding studies with a sample size of 10 or less, and (3) excluding studies published before the end of 2010. All *P* values were 2-sided and a *P* value of less than 0.05 was deemed statistically significant.

## Results

### Characteristics of included studies

**Fig. 1:**
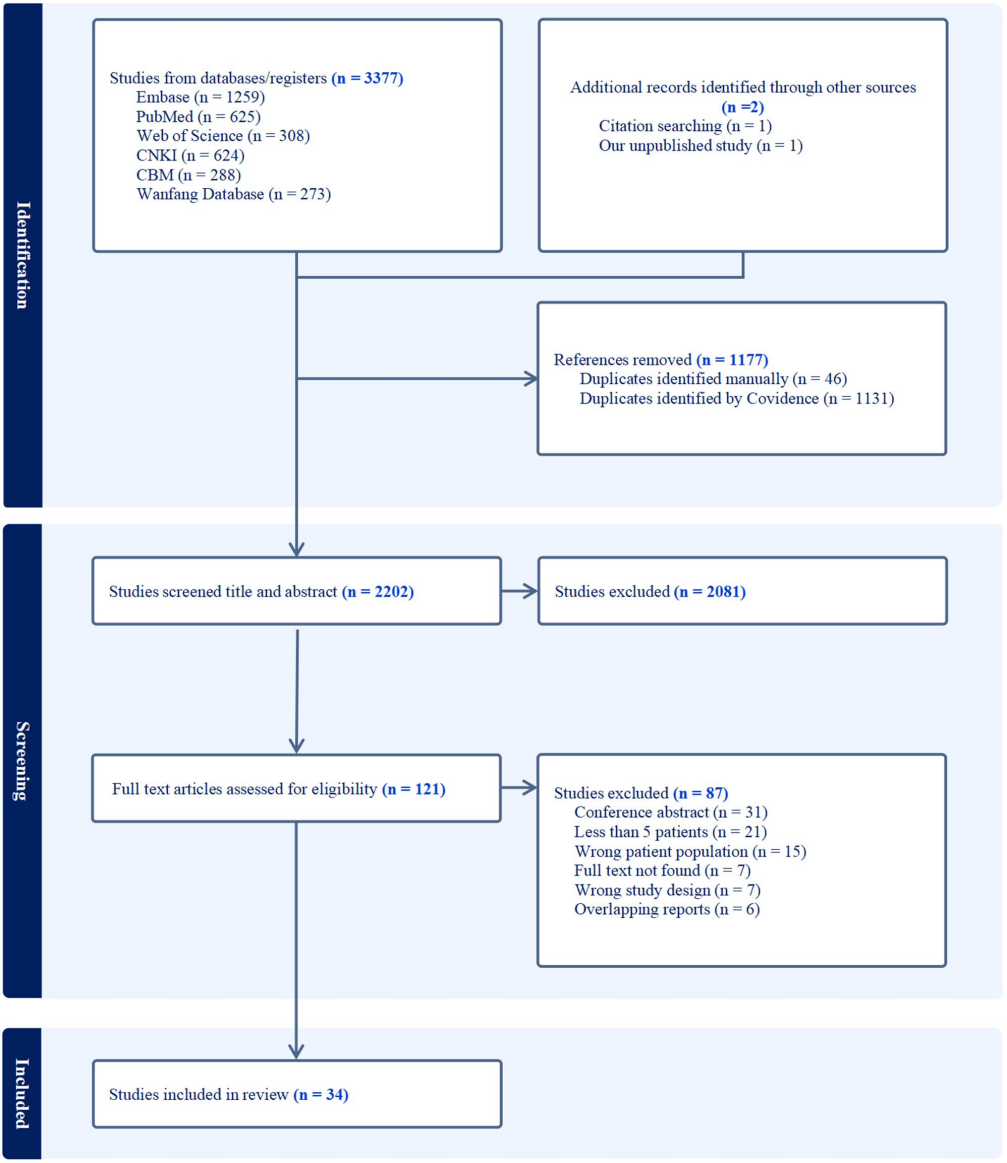
Flow diagram of study selection according to Preferred Reporting Items for Systematic Reviews and Meta-Analyses (PRISMA) guidelines

A total of 3379 studies were identified. Of all identified studies, 1177 duplicates were removed. A further 2081 were excluded after title and abstract screening. Subsequently, the full texts of 121 studies were reviewed and a further 87 articles were removed based on inclusion and exclusion criteria. Ultimately, 34 eligible studies were included in the meta-analyses with a total of 609 late-onset MADD patients (Fig. 1).

The study design of all included studies was case series and their characteristics are shown in Table 1. Most studies were performed in tertiary care centers (32 [94.12%]). Study period ranged from 1991 to 2022. Twenty-three studies were conducted in East Asia, 4 in West Asia, and 7 in Europe. All studies were carried out in countries with high (22 [64.71%]) or very high (12 [35.29%]) HDI tiers. The sample size of selected studies ranged from 5 to 110.

**Table 1:** Summary of all the selected studies

First author	Publication year	Study period	Country	Sample size	HDI	MFR of general population
Shimizu [e47]	1991	NR	Japan	5	Very high	0.95:1
Russell [e48]	2003	NR	Italy	7	Very high	0.95:1
Yang [e49]	2004	2000–2003	China	7	High	1.06:1
Olsen [19]	2007	1998–2006	Denmark	15	Very high	0.99:1
Er [e50]	2010	NR	China	9	High	1.06:1
Lan [e51]	2010	2001–2008	China	9	High	1.06:1
Wang [8]	2011	NR	China	56	High	1.06:1
Wang [e52]	2011	2003–2010	China	24	High	1.06:1
Xi [e53]	2011	2005–2010	China	31	High	1.06:1
Liu [e54]	2012	NR	China	8	High	1.06:1
Fang [e55]	2014	2008–2013	China	5	High	1.06:1
Zhu [9]	2014	2011–2013	China	13	High	1.06:1
Béhin [20]	2016	NR	France	13	Very high	0.93:1
Liu [e56]	2016	2012–2015	China	28	High	1.06:1
Olsen [e57]	2016	NR	Denmark	7	Very high	0.99:1
Angelini [e58]	2018	NR	Italy	6	Very high	0.95:1
Zhao [15]	2018	2013–2016	China	25	High	1.06:1
Henriques [e59]	2019	NR	Portugal	5	Very high	0.95:1
Hong [17]	2019	2014–2018	China	25	High	1.06:1
Nilipour [18]	2020	2011–2016	Iran	19	High	0.99:1
Sun [e60]	2020	2012–2017	China	5	High	1.06:1
Yildiz [e61]	2020	NR	Turkey	20	Very high	1:1
Yuan [e62]	2020	2009–2019	China	15	High	1.06:1
Ali [e63]	2021	2009–2020	United Arab Emirates	11	Very high	2.26:1
Kuo [e64]	2021	1998–2018	China	6	High	1.06:1
Staretz-Chacham [e65]	2021	NR	Israel	5	Very high	1:1
Tang [e66]	2021	2018–2021	China	5	High	1.06:1
Liu [e67]	2022	2009–2021	China	26	High	1.06:1
Lupica [e68]	2022	2001–2021	Italy	10	Very high	0.95:1
Wen [5]	2022	1995–2019	China	110	High	1.06:1
Yamada [e69]	2022	1997–2020	Japan	22	Very high	0.95:1
Zhang [e70]	2022	2005–2020	China	31	High	1.06:1
Zheng [16]	2022	2019–2021	China	13	High	1.06:1
Zhang [6]	2023	2016–2022	China	13	High	1.06:1

Legend: HDI, Human Development Index; MFR, male to female ratio; NR, not reported

### Risk of bias of included studies

The bias risk assessment using the JBI checklists showed that all of the included studies fell into the JBI low-risk category (Supplementary Table 2).

### Sex distribution in patients with late-onset MADD

All 34 studies reported the number of male and female patients with late-onset MADD. According to the results of the random-effects model, the pooled percentage of male patients was 58% (95% CI, 54-63%). There was a low variation in sex distribution across studies (*I*^*2*^ = 2.99%; *P* = 0.418). The results of sensitivity analyses did not indicate a substantial difference in the proportions (Table 2).

**Table 2:** Sensitivity analyses after excluding studies with a relative high risk of bias, with a small sample size, or published before 2011

Characteristic	Number of studies combined	Total number of patients	Proportion from random-effects meta-analysis (95% CI)	*I*^2^, %
JBI score				
≥ 90%	17	384	61% (55%, 66%)	0.00%
Sample size				
> 10	19	510	57% (52%, 62%)	16.29%
Years of publication				
2011–2023	28	557	59% (54%, 63%)	0.00%

Legend: JBI, Joanna Briggs Institute

### Sex differences in age of onset, diagnostic delay, serum CK at diagnosis, and allelic frequencies of the hotspot variants in ETFDH gene

A total of 33, 23, 19 and 27 studies reported age of onset, diagnostic delay, serum CK at diagnosis, and disease-associated mutations of each patient with late-onset MADD, respectively. The weighted means of onset ages (*P* = 0.467), diagnostic delay (*P* = 0.597), serum CK (*P* = 0.923) (Supplementary Fig. 1), and ORs for allelic frequencies of the 3 hotspot variants in *ETFDH* gene (*P* > 0.05) (Supplementary Fig. 2) were similar between male and female patients.

### Influences on the MFR

The univariable meta-regressions revealed that ethnic group (*P* < 0.001), onset age (*P* = 0.026), and HDI (*P* = 0.003) were each individually associated with the MFR in late-onset MADD. However, once we identified and controlled for confounders, only ethnic group remained a significant predictor (*P* = 0.045) (Table 3).

**Table 3:** Univariable meta-regressions model for investigating influence on the male frequency of late-onset MADD

Variants	Number of tudies	Unadjusted association with male frequency of late-onset MADD				Adjusted association with male frequency of late-onset MADD			
		Coef	Standard Error	*P* value	95% CI	Coef	Standard Error	*P* value	95% CI
Ethnic group*	34	0.350	0.100	< 0.001	0.154–0.547	0.316	0.158	0.045	0.007–0.625
Risk of bias	34	0.940	0.495	0.058	-0.030-1.910	-	-	-	-
Onset age	33	0.010	0.005	0.026	0.001–0.019	0.005	0.006	0.392	-0.006-0.016
MFR of general population of country	34	-0.077	0.242	0.750	-0.551-0.397	-	-	-	-
HDI of country	34	0.289	0.098	0.003	0.097–0.481	-0.003	0.173	0.987	-0.342-0.337

Legend: MADD, multiple acyl-CoA dehydrogenase deficiency; MFR, male to female ratio; Coef, coefficient; CI, confidence interval; HDI, Human Development Index

*Ethnic group: East-Asians or non-East-Asians

**Fig. 2:**
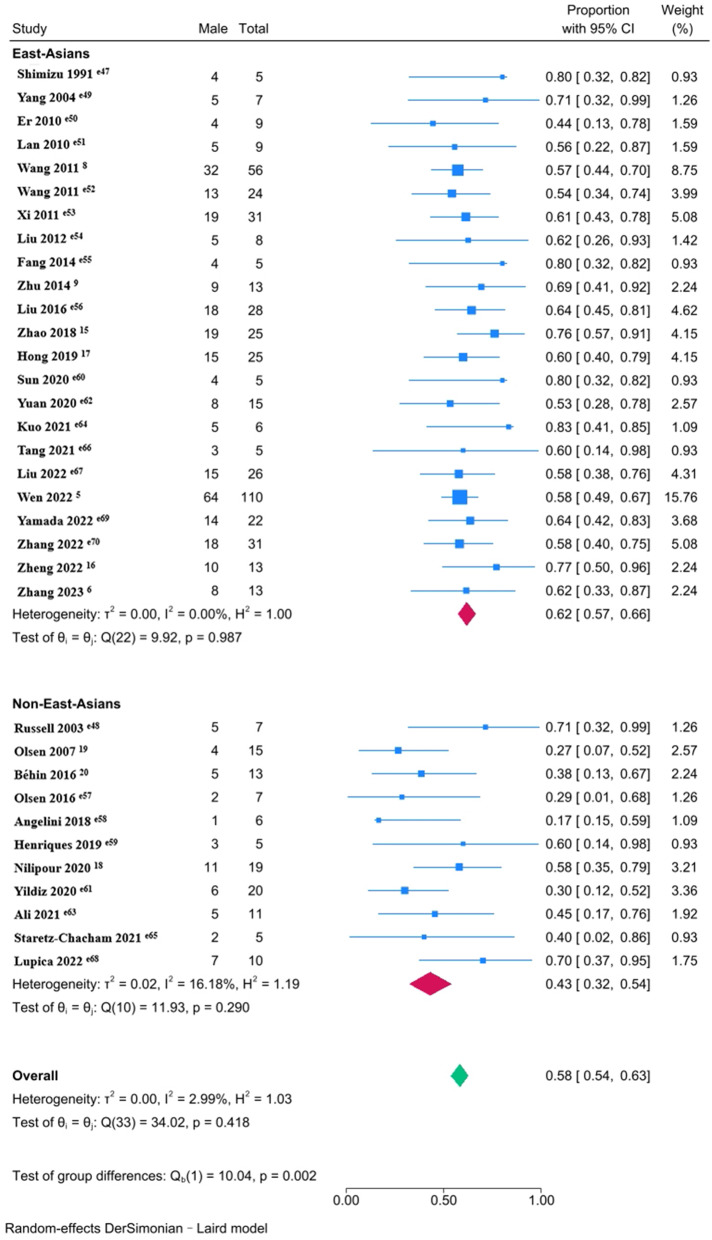
Forest plots for the percentage of male patients with late-onset MADD stratified by ethnic groups (East-Asians vs. non-East-Asians)

The subgroup meta-analyses demonstrated that East-Asians had a higher percentage of male patients with late-onset MADD (62% [95% CI, 57-66%]) than non-East-Asians (43% [95% CI, 32-54%]; *P* = 0.002) (Fig. 2). Although onset age (*P* = 0.202) and diagnostic delay (*P* = 0.298) were similar between the two ethnic groups, serum CK at diagnosis was significantly higher in non-East-Asian patients (*P* = 0.049), indicating a more severe phenotype (Supplementary Fig. 3). The 3 hotspot variants in *ETFDH* gene were more common in East-Asian patients (*P* < 0.05) (Supplementary Fig. 4).

Another subgroup meta-analysis for different onset age groups showed that the male proportion was 51% (95% CI, 39-62%) for patients included in studies with average disease-onset age < 20 years old, and 60% (95% CI, 52-68%) for patients included in studies with average disease-onset age ≥ 20 years old.

### Publication bias

**Fig. 3:**
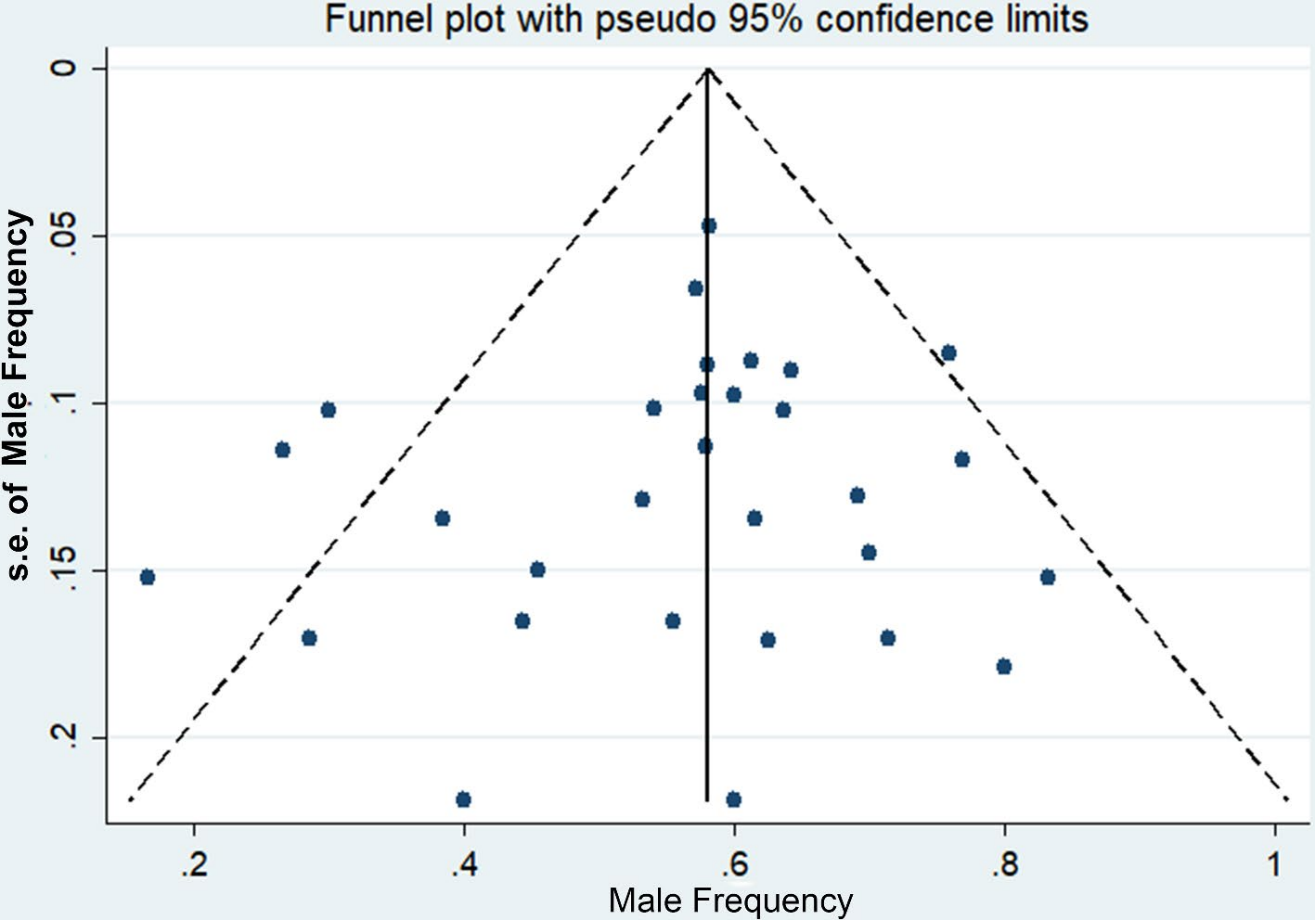
Funnel plot for the male frequency of late-onset MADD

We investigated the association of potential publication bias with MFR of late-onset MADD by creating a funnel plot from included studies. The vertical axis of the funnel plot represents standard error of effect size (percentage of male patients) while the horizontal axis represents effect size. The funnel plot was symmetric (Fig. 3) and Egger regression test provided no evidence of substantial publication bias (t = − 0.11; *P* = 0.917).

## Discussion

To our knowledge, this is the first systematic review and meta-analysis investigating the MFR in late-onset MADD. A total of 34 studies enrolling 609 patients with late-onset MADD were included. The meta-analyses revealed that male patients with late-onset MADD significantly outnumbered female patients, and the meta-regression showed that ethnic group had an impact on the MFR.

Autosomal recessive disorders usually affect men and women equally [14]. For example, approximately half of the patients with Pompe disease are female in China (49.2%) [27] and worldwide (51.2%) [28]. Also, the MFR of spinal muscular atrophy (SMA) is nearly 1:1 in two large SMA registries, the TREAT-NMD Global SMA Patient Registry (2263/2261) [29] and the Cure SMA database (4528/4526) [30]. However, the overall pooled male frequency of late-onset MADD was 58% (95% CI, 54-63%) in our study, although the disease is inherited in an autosomal recessive manner. The results suggest that males may be more vulnerable to late-onset MADD than females.

Although there is diversity in economic, geographical, and social conditions across countries, it seems that these parameters do not influence the frequency of male patients with MADD. In our included studies, there were both more male patients in Japan [e47, e69] and Italy [e48, e68], which is the developed Asian country and developed European country, respectively. In contrast, in other Asian developing countries, such as United Arab Emirates [e63] and Turkey [e61], there were more female patients with MADD than male patients. In terms of culture, the male to female ratios of general population of different countries are similar and close to 1 except for United Arab Emirates, although the cultures vary significantly from country to country.

Metabolic stress, such as high fat diet, excessive physical training, and using anti-obesity drugs, is essential to the disease onset, which works possibly by increasing the activity of fatty acid β-oxidation [7, 10, 31] and the subsequent increase in production of reactive oxygen species (ROS) [32, 33]. We hypothesize that the effects of gender on late-onset MADD is mediated by sex differences in fat metabolism. Men have more muscle mass than women, and the basal fat oxidation is higher in men than in women [34, 35]. Male sex can therefore lower the threshold of developing the disease.

Gonadal hormones are a major determinant of sex differences in fat metabolism [36]. Androgens are implicated to regulate male and female energy balance, of which testosterone is the most important biologically relevant form [37, 38]. In men and women, testosterone deficiency leads to decreased muscle mass and testosterone supplementation results in increases in both muscle mass and strength [39–41]. In male hypogonadism, fatty acids are not efficiently burned by β-oxidation and body fat mass is increased, suggesting a possible role of testosterone [42, 43]. On the contrary, testosterone substitution of hypogonadal men prevents the increase in body mass index [42], and testosterone can induce energy expenditure in skeletal muscle via enhancing mitochondrial biogenesis and fatty acid β-oxidation in male mice [44]. In addition, testosterone undecanoate treatment, usually used in people transitioning from female-to-male, can induce an increase in ROS production [45]. Furthermore, it is well known that the sex hormones vary greatly across age and reach peak secretion between the age of 20 and 50 s [26]. Consistent with this changing trend, our results suggested that the pooled percentage of male patients was significantly higher in patients included in studies with average disease-onset age ≥ 20 years old, while late-onset MADD affected male and female equally in patients included studies with average disease-onset age < 20 years old. As a result, higher levels of androgens may contribute to the susceptibility of late-onset MADD in males, but more work is required to elicit the mechanism of action.

As male sex is supposed to play a role in the presentation of late-onset MADD, we performed additional meta-analyses to investigate the differences of clinical characteristic between male and female patients. However, men and women were comparable across the variables, including onset age, diagnostic delay, serum CK at diagnosis, and allelic frequencies of the 3 hotspot variants in *ETFDH* gene. It appears that gender does not affect the severity of the disease and mutation spectrum is similar between the sexes.

To explore the factors influencing the MFR in late-onset MADD, we conducted meta-regression and subgroup meta-analyses, which indicated an effect of ethnic group on MFR. Late-onset MADD affected male and female equally in non-East-Asian patients. We speculate the differences between the ethnic groups are caused by different mutation spectrum in *ETFDH*. Our results verified that non-East-Asians with late-onset MADD rarely carried the 3 hotspot variants in *ETFDH*, which were common in East-Asian patients. There is a clear relationship between *ETFDH* genotype and phenotype in patients with MADD [46]. Non-East-Asian patients exhibited a more severe phenotype, as reflected by a higher level of serum CK. In this case, the effect of gender on the disease onset may be overshadowed by the mutations in *ETFDH* leading to fewer residual enzyme activity.

Our study has several limitations. Because of the eligibility criteria, some studies were not included in our review. For instance, if there was an overlap of participants between two studies, the one with smaller sample size was excluded. However, the lack of primary information in the data sources makes it impossible to separate them. In addition, most included studies were hospital-based case series, which lead to selection bias. Presumably, there may be mild patients who do not go to hospitals. Furthermore, the male-to-female odds ratio could not be calculated due to the design of the included studies.

In conclusion, the results of the present study demonstrated that patients with late-onset MADD were more common in male than female patients. Male sex may be a risk factor for the disease. However, given that all included studies were case series, further studies are needed to support the veracity of our findings.

### Electronic supplementary material

Below is the link to the electronic supplementary material.

**Supplementary Figure 1.** Forest plots for the weighted means of onset age (a), diagnostic delay (b), and serum CK (c) in male vs female patients with late-onset MADD.

**Supplementary Figure 2.** Forest plots for the frequencies of the 3 hotspot mutations in *ETFDH* gene (c.250G > A [a], c.770 A > G [b], and c.1227 A > C [c]) in male vs. female patients with late-onset MADD.

**Supplementary Figure 3.** Forest plots for the weighted means of onset age (a), diagnostic delay (b), and serum CK (c) in patients with late-onset MADD stratified by ethnic groups (East-Asians vs. non-East-Asians).

**Supplementary Figure 4.** Forest plots for the frequencies of the 3 hotspot mutations in *ETFDH* gene (c.250G > A [a], c.770 A > G [b], and c.1227 A > C [c]) in patients with late-onset MADD stratified by ethnic groups (East-Asians vs. non-East-Asians).

Supplementary Material 5

Supplementary Material 6

Supplementary Material 7

## Data Availability

Data supporting the findings of this study are available within the paper and its Supplementary Information.
