# Supplementary figures and images for: The male-to-female ratio in late-onset multiple acyl-CoA dehydrogenase deficiency: a systematic review and meta-analysis
Source: Orphanet J Rare Dis. 2024 Feb 16;19:72. doi: 10.1186/s13023-024-03072-6 (PMC10873946; doi:10.1186/s13023-024-03072-6)

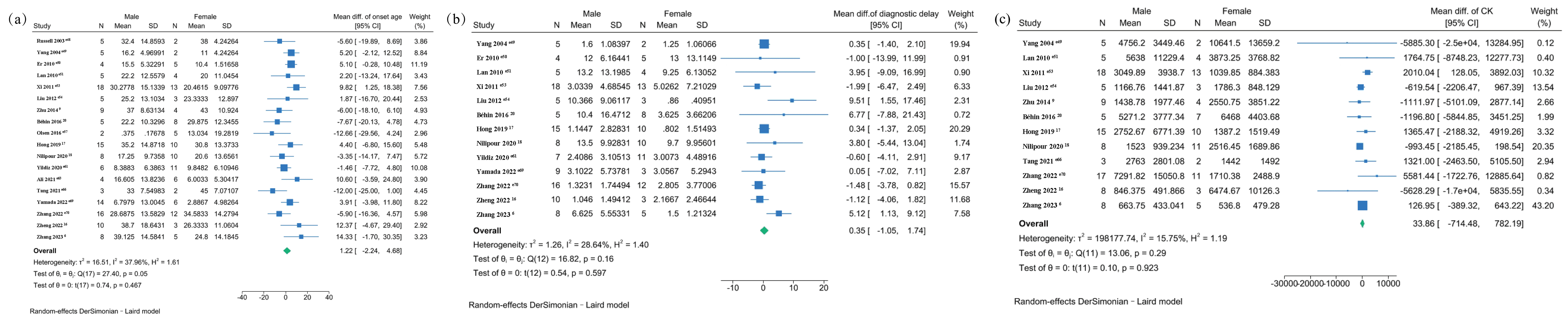

Supplement: Supplementary file 1 — Supplementary Figure 1. Forest plots for the weighted means of onset age (a), diagnostic delay (b), and serum CK (c) in male vs female patients with late-onset MADD. [file 13023_2024_3072_MOESM1_ESM.jpg]

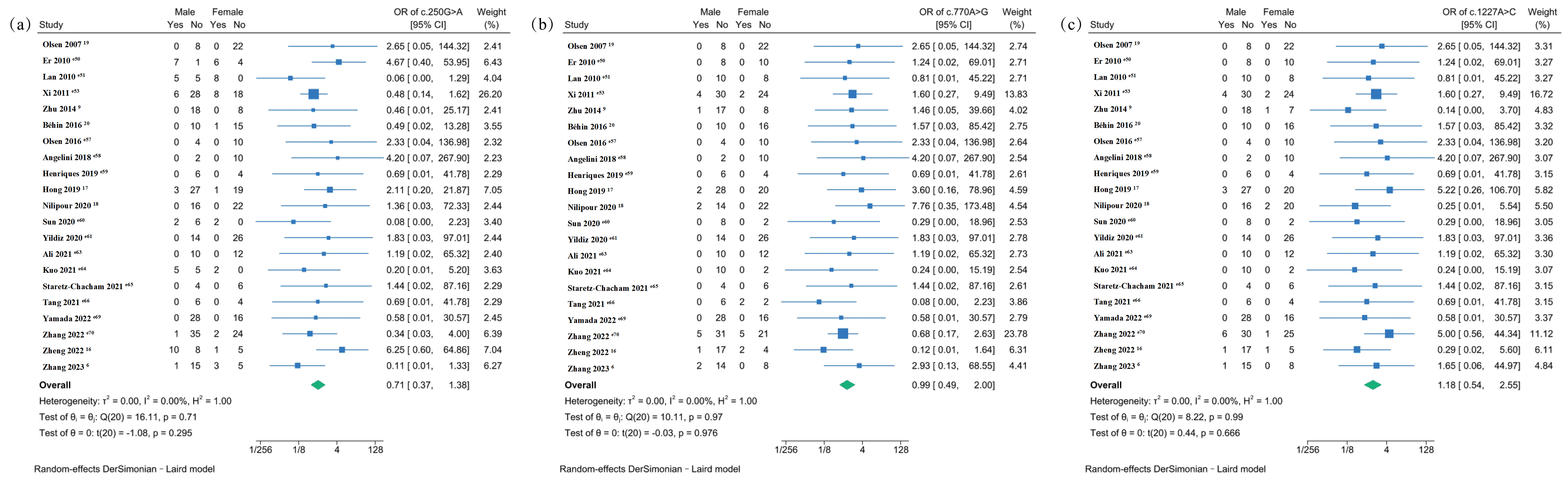

Supplement: Supplementary file 2 — Supplementary Figure 2. Forest plots for the frequencies of the 3 hotspot mutations in ETFDH gene (c.250G > A [a], c.770 A > G [b], and c.1227 A > C [c]) in male vs. female patients with late-onset MADD. [file 13023_2024_3072_MOESM2_ESM.jpg]

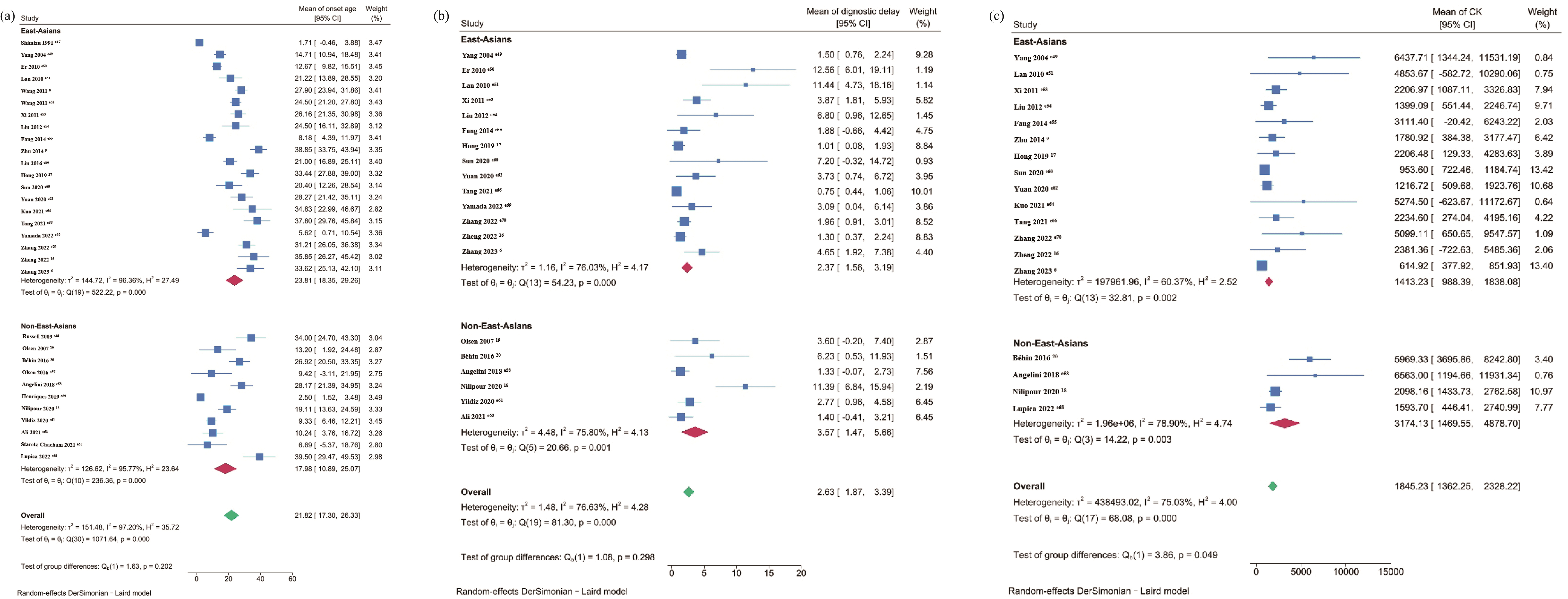

Supplement: Supplementary file 3 — Supplementary Figure 3. Forest plots for the weighted means of onset age (a), diagnostic delay (b), and serum CK (c) in patients with late-onset MADD stratified by ethnic groups (East-Asians vs. non-East-Asians). [file 13023_2024_3072_MOESM3_ESM.jpg]

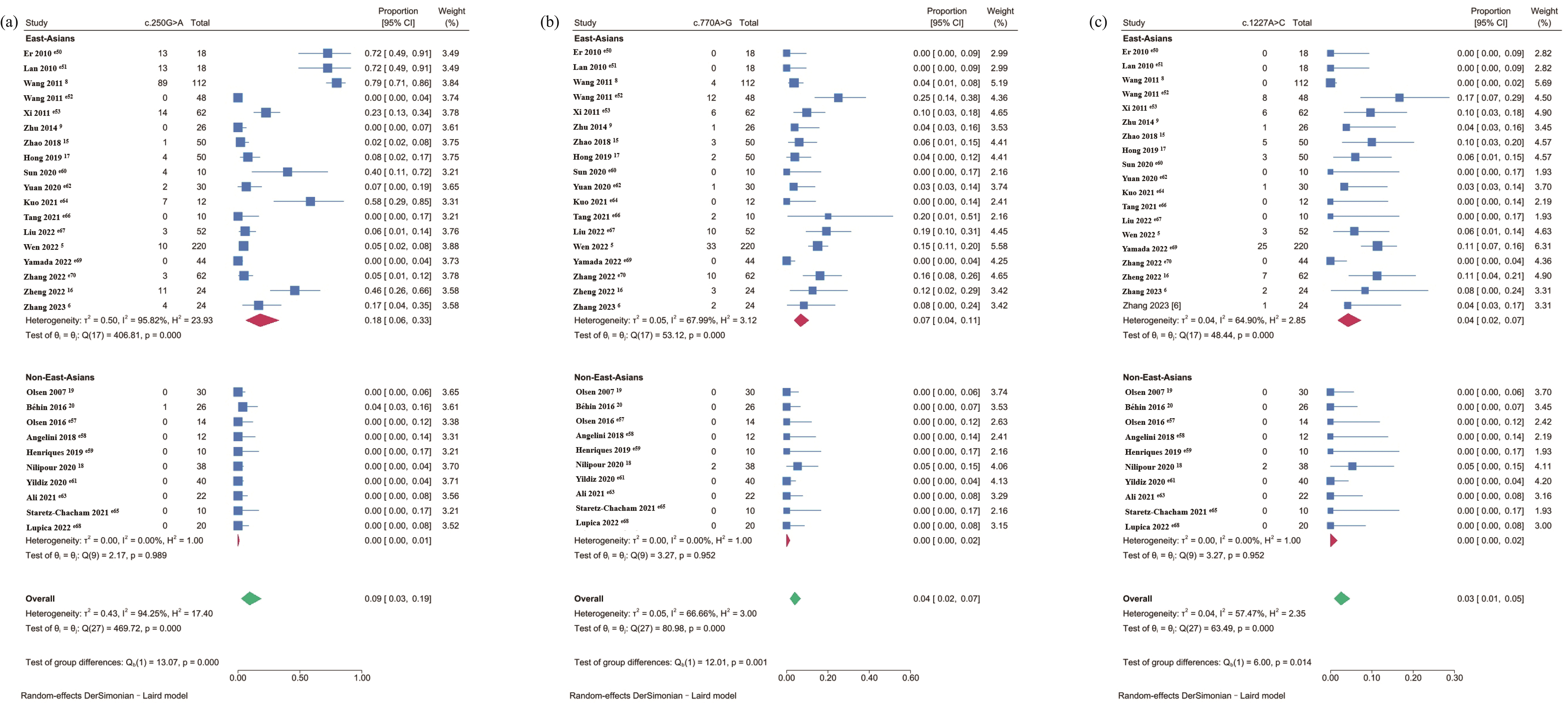

Supplement: Supplementary file 4 — Supplementary Figure 4. Forest plots for the frequencies of the 3 hotspot mutations in ETFDH gene (c.250G > A [a], c.770 A > G [b], and c.1227 A > C [c]) in patients with late-onset MADD stratified by ethnic groups (East-Asians vs. non-East-Asians). [file 13023_2024_3072_MOESM4_ESM.jpg]
