# Supplementary material for: The male-to-female ratio in late-onset multiple acyl-CoA dehydrogenase deficiency: a systematic review and meta-analysis
Source: Orphanet J Rare Dis. 2024 Feb 16;19:72. doi: 10.1186/s13023-024-03072-6 (PMC10873946; doi:10.1186/s13023-024-03072-6)
Supplement: Supplementary file 6 — Supplementary Material 6 [file 13023_2024_3072_MOESM6_ESM.docx]

**Supplementary references**

e47. Shimizu, N., et al., *Mass spectrometric analysis of metabolite excretion in five Japanese patients with the late-onset form of glutaric aciduria type II.* Biological mass spectrometry, 1991. **20**(8): p. 479-83. <https://doi.org/10.1002/bms.1200200808>

e48. Russell, A., et al., *Decreased fatty acid beta-oxidation in riboflavin-responsive, multiple acylcoenzyme A dehydrogenase-deficient patients is associated with an increase in uncoupling protein-3.* The Journal of clinical endocrinology and metabolism, 2003. **88**(12): p. 5921-6. <https://doi.org/10.1210/jc.2003-030885>

e49. Yang, Y., KIMURA Masahiko,Yuan Yun, Qian Ning, Liu Xue-qin, Zhang Yue-hua, Bao Xin-hua, Wu Ye, Sun Fang, Song Jin-qing, and Y.G.S. HASEGAWA Yuki, SHIGEMA TSU Yousuke, QIN Jiong, WU Xi ru, *Clinical and biochemical diagnostic and therapeutic survey of seven patients with lipid storage myopathy due to glutaric aciduria type Ⅱ(in Chinese).* Chin J Neurol, 2004. **Vol37**.

e50. Er, T., et al., *High resolution melting analysis facilitates mutation screening of ETFDH gene: applications in riboflavin-responsive multiple acyl-CoA dehydrogenase deficiency.* Clinica chimica acta; international journal of clinical chemistry, 2010. **411**: p. 690-9. <https://doi.org/10.1016/j.cca.2010.01.033>

e51. Lan, M., et al., *High frequency of ETFDH c.250G>A mutation in Taiwanese patients with late-onset lipid storage myopathy.* Clinical genetics, 2010. **78**(6): p. 565-9. <https://doi.org/10.1111/j.1399-0004.2010.01421.x>

e52. Wang Yun, Z.D.-h., Hong Dao-jun, Wang Zhaoxia，Yuan Yun, *Hot spot mutations in electron transfer flavoprotein dehydrogenase gene of riboflavin responsive lipidstorage myopathy in 20 Chinese families(in Chinese).* Chin J Neurol, 2011. **Vol. 44**. <https://doi.org/10.3760/cma.j.issn.1006-7876.2011.05.005>

e53. Xi Jian-ying, L.J.-h., Zhao Chong-bo, Lin Jie, Luo Su-shan, Zhu Wen-hua, Qiao Kai, Huang Jun, Wang Yin *Clinical features and electron transfer flavoprotein dehydrogenase gene mutation analysis in 35 Chinese patients with lipid storage myopathy(in Chinese).* Chin J Neurol, 2011. **Vol. 44**. <https://doi.org/10.3760/cma.j.issn,1006-7876.2011.05.006>

e54. Liu Peng, W.Q., Pu Chuanqiang *The study of diagnosis of MADD throuth screening urine organic acids(in Chinese).* Journal of Logistics University of CAPF（Medical Sciences）, 2012. **vol.21** <https://doi.org/10.3969/j.issn.2095-3720.2012.03.011>

e55. Fang Runtao, L.J., Yang Yanling, Jin Yiwen, Ji Taoyun, Xiong Hui, Wang Shuang, Zhang Yuehua, Bao Xinhua, Zhang Yao, Qin Jiong, Chang Xingzhi, *Clinical, pathological and molecular genetic analysis of pediatric lipid storage myopathy(in Chinese).* Chin J Appl Clin Pediatr, 2014. **Vol29**. <https://doi.org/10.3760/cma.j.issn.2095-> 428X.2014.14.014

e56. Liu, X., et al., *Skeletal Muscle Magnetic Resonance Imaging of the Lower Limbs in Late-onset Lipid Storage Myopathy with Electron Transfer Flavoprotein Dehydrogenase Gene Mutations.* Chinese medical journal, 2016. **129**(12): p. 1425-31. <https://doi.org/10.4103/0366-6999.183423>

e57. Olsen, R., et al., *Riboflavin-Responsive and -Non-responsive Mutations in FAD Synthase Cause Multiple Acyl-CoA Dehydrogenase and Combined Respiratory-Chain Deficiency.* American journal of human genetics, 2016. **98**(6): p. 1130-1145. <https://doi.org/10.1016/j.ajhg.2016.04.006>

e58. Angelini, C., D. Tavian, and S. Missaglia, *Heterogeneous Phenotypes in Lipid Storage Myopathy Due to ETFDH Gene Mutations.* JIMD reports, 2018. **38**: p. 33-40. <https://doi.org/10.1007/8904_2017_27>

e59. Henriques, B., et al., *Molecular and Clinical Investigations on Portuguese Patients with Multiple acyl-CoA Dehydrogenase Deficiency.* Current molecular medicine, 2019. **19**(7): p. 487-493. <https://doi.org/10.2174/1566524019666190507114748>

e60. Sun Yi-ming, C.J.-q., Li Jing, Li Huan, Zhang Cheng, *The clinical and genetical heterogeneity of riboflavin - responsive multiple acylcoenzyme A dehydrogenase deficiency.* Chin J Contemp Neurol Neurosurg, 2020. **Vol. 20**. <https://doi.org/10.3969/i.issn.1672-6731.2020.06.011>

e61. Yıldız, Y., et al., *Determinants of Riboflavin Responsiveness in Multiple Acyl-CoA Dehydrogenase Deficiency.* Pediatric neurology, 2019. **99**: p. 69-75. <https://doi.org/10.1016/j.pediatrneurol.2019.06.015>

e62. Yuan Jiao, L.Q., Lu Ke, Zeng Qianqian, Zhou Jinxia, Bi Fangfang, *Clinical, pathological, and genetic features of riboflavin responsive multiple acyl-CoA dehydrogenation deficiency caused by electron transfer flavoprotein dehydrogenase mutation(in Chinese).* Journal of International Neurology and Neurosurgery, 2020. **47(3)**. <https://doi.org/>

10.16636/j.cnki.jinn.2020.03.019

e63. Ali, A., et al., *Clinical, Biochemical, and Genetic Heterogeneity in Glutaric Aciduria Type II Patients.* Genes, 2021. **12**(9). <https://doi.org/10.3390/genes12091334>

e64. Kuo, Y., et al., *A systematic review of late-onset and very-late-onset multiple acyl-coenzyme A dehydrogenase deficiency: Cohort analysis and patient report from Taiwan.* Neuromuscular disorders: NMD, 2021. **31**(3): p. 218-225. <https://doi.org/10.1016/j.nmd.2021.01.006>

e65. Staretz-Chacham, O., et al., *Multiple Acyl-CoA Dehydrogenase Deficiency with Variable Presentation Due to a Homozygous Mutation in a Bedouin Tribe.* Genes, 2021. **12**(8). <https://doi.org/10.3390/genes12081140>

e66. Tang, Z., et al., *ETFDHClinical Presentations and Genetic Characteristics of Late-Onset MADD Due to Mutations in Five Patients: A Case Series.* Frontiers in neurology, 2021. **12**: p. 747360. <https://doi.org/10.3389/fneur.2021.747360>

e67. Liu Haiyan, F.J., Ma Mingming , Qu Qianqian, Qian Qi, Cui Wenhao, Zhang Yan, Lyu Haidong, *Clinical characteristics and electron transfer flavoprotein dehydrogenase genetic mutations in 26 patients with riboflavin reactive lipid deposition myopathy(in Chinese).* Chin J Neuromed, 2022. **Vol. 21**. <https://doi.org/10.3760/cma.j.cn115354-20211118-00748>

e68. Lupica, A., et al., *Diagnostic Challenges in Late Onset Multiple Acyl-CoA Dehydrogenase Deficiency: Clinical, Morphological, and Genetic Aspects.* Frontiers in neurology, 2022. **13**: p. 815523. <https://doi.org/10.3389/fneur.2022.815523>

e69. Yamada, K., et al., *ETFDHClinical and molecular investigation of 37 Japanese patients with multiple acyl-CoA dehydrogenase deficiency: p.Y507D in , a common Japanese variant, causes a mortal phenotype.* Molecular genetics and metabolism reports, 2022. **33**: p. 100940. <https://doi.org/10.1016/j.ymgmr.2022.100940>

e70. Zhang, J., et al., *Characterization of 31 Patients with Riboflavin-Responsive Multiple acyl-CoA Dehydrogenase Deficiency.* Balkan medical journal, 2022. **39**(4): p. 290-296. <https://doi.org/10.4274/balkanmedj.galenos.2022.2022-1-127>
